# Beyond the Checkpoint: Severe Axitinib-induced Liver Injury

**DOI:** 10.14309/crj.0000000000001177

**Published:** 2023-11-04

**Authors:** Yee Hui Yeo, Walid Ayoub, Ju Dong Yang, Alexander Kuo, Hirsh D. Trivedi

**Affiliations:** 1 Karsh Division of Gastroenterology and Hepatology, Department of Medicine, Cedars-Sinai Medical Center, Los Angeles, CA

## Abstract

Understanding the potential adverse effects associated with oncological treatments is crucial in the clinical care of patients with cancer. We describe the first case report delineating severe acute liver injury secondary to axitinib. This is a case of metastatic renal cell carcinoma treated with axitinib and pembrolizumab, complicated by a severe axitinib-induced liver injury, characterized by significant elevations of hepatocellular and cholestatic liver enzymes during initial treatment and rechallenge of axitinib. Remarkably, the liver chemistries normalized upon discontinuation of the medication.

## INTRODUCTION

Drug-induced liver injury (DILI) is a common complication of oncologic medication.^1,2^ Many chemotherapeutic agents, targeted therapies, and, more recently, immunotherapy can contribute to DILI.^3^ Understanding the implications of hepatotoxicity in the context of potentially life-saving therapy is paramount when managing patients with malignancy.

Trials and case reports suggest that molecular targeted therapy, including tyrosine kinase inhibitor and BRAF kinase inhibitor, can result in DILI.^4^ Vascular endothelial growth factor (VEGF) inhibitors, known for their hepatotoxicity,^5^ carry a black box warning.^4^ However, given the rise of immunotherapy-induced drug injuries, the injury caused by VEGF inhibitors could be masked when coadministered with immunotherapies. The interplay between VEGF inhibitors and immunotherapies could further complicate the detection of DILI.^6^ Therefore, it is imperative to recognize and address the potential hepatotoxicity. We present axitinib-induced severe acute liver injury of a mixed hepatocellular and cholestatic pattern in a patient on pembrolizumab for metastatic renal cell carcinoma.

## CASE REPORT

This is a 61-year-old man with a history of metastatic renal cell carcinoma with bone metastasis being treated with denosumab. The renal cell carcinoma was diagnosed 6 months before presentation, which led to a left nephrectomy.

**Figure 1.**
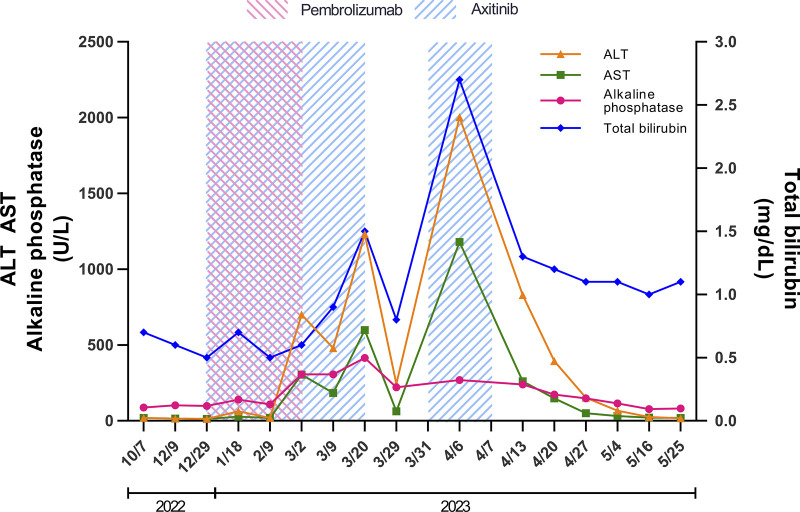
The trend of liver chemistries from before the use through after the discontinuation of axitinib. The pink shaded area denotes the period with pembrolizumab use. The blue shaded area denotes the period with axitinib use. Overlapped area denotes the use of both medications. ALT, alanine transaminase; AST, aspartate transaminase.

Two months after the nephrectomy, the patient commenced an immunotherapy/targeted therapy regimen of axitinib and pembrolizumab, alongside a course of radiation therapy, which concluded with 10 sessions. However, the patient's fourth cycle of immunotherapy/targeted therapy was interrupted 2 months after the initiation of the therapy because of elevated liver enzymes: alanine transaminase (ALT) 702 U/L, aspartate transaminase (AST) 306 U/L, and alkaline phosphatase (ALP) 308 U/L, all increased from their baseline normal range (Figure 1). The total bilirubin level was normal. The patient was without any discomfort or pain and denied any symptoms. He denied taking any new medications, nutrition supplements, or herbs. The workup for chronic liver disease (viral hepatitis, autoimmune, and hereditary liver diseases) did not reveal other potential etiologies of the elevated liver chemistries. Abdominal and pelvic computed tomography with contrast performed while he was on immunotherapy/targeted therapy showed only mild fatty infiltration in the liver. Owing to concern for possible checkpoint inhibitor hepatitis, pembrolizumab was held while axitinib was continued.

Subsequent laboratory results showed a persistent increase in liver chemistries, with AST, ALT, ALP, and total bilirubin surging to 416 U/L, 1233 U/L, 598 U/L, and 1.5 mg/dL, respectively, despite having stopped pembrolizumab (Figure 1). Axitinib was, therefore, also discontinued, followed by a notable improvement in liver enzymes, with AST, ALT, ALP, and total bilirubin decreasing to 222 U/L, 245 U/L, 64 U/L, and 0.8 mg/dL, respectively. Axitinib was eventually reintroduced while pembrolizumab remained on hold. Unfortunately, his liver enzymes subsequently rose again (AST 1180 U/L, ALT 2003 U/L, ALP 270 U/L, and total bilirubin 2.7 mg/dL). Given the severe liver injury, now with a cholestatic component, axitinib was again discontinued and the liver enzymes again normalized. During this period, there was no change in other medications. Liver biopsy was not performed because his liver chemistries worsened after rechallenging with axitinib and completely normalized after its discontinuation, making our diagnosis of axitinib-induced liver injury certain.

## DISCUSSION

We present a case with severe axitinib-related liver injury characterized by marked elevations of hepatocellular and cholestatic liver enzymes during initial treatment and rechallenge of axitinib with subsequent normalization of liver chemistries after the discontinuation of the medication. To our knowledge, this is the first case report of severe acute liver injury from axitinib with cholestatic hepatitis. Understanding its risk profile, particularly while taking concurrent immunotherapy, is paramount in the overall management of those with malignancy. In our case, discontinuing the immunotherapy, pembrolizumab did not affect the level of his liver chemistries, pointing toward axitinib as the primary causative agent. This case underscores the complexities of managing oncological therapy-related liver injuries, highlighting the need for meticulous monitoring and proactive management to achieve early recognition, prevent irreversible hepatic damage, and optimize patient outcomes.

Axitinib, a selective inhibitor of the VEGFR,^7^ showed in a phase III trial the most common grade ≥3 adverse events were hypertension, diarrhea, and fatigue; 32% had elevated thyroid-stimulating hormone, necessitating thyroid hormone management.^8^ A phase 1b trial with axitinib and pembrolizumab reported relatively mild elevations in liver enzymes (AST and ALT)^9^ while the proportion of grade 3 or 4 liver enzyme elevation was higher in the KEYNOTE-426 trials, which used pembrolizumab and axitinib as the first-line treatment in patients with advanced renal cell carcinoma.^10^ Currently, axitinib is categorized as a suspected, but unproven, hepatotoxic agent according to the LiverTox website.^11^ This is the first case report that showed a grade 4 liver enzyme elevation (AST and ALT >10.0 times upper limit of normal [ULN]) after axitinib administration. In addition, the liver injury after the rechallenge of axitinib, in this case, has met the Hy law criterion, which is defined as concurrent elevations of ALT (>3× ULN) and total bilirubin (>2× ULN) without initial evidence of cholestasis. The Hy law is a sensitive and specific predictor for a medication to trigger severe hepatotoxicity^12^ and potentially indicates a need for liver transplantation in 10% of such cases.^13^ Notably, according to the previous consensus, both pembrolizumab and axitinib should be discontinued permanently when a grade 4 hepatotoxicity is found.^14^

VEGF is involved in numerous physiological processes, including endothelial cell growth and migration.^15,16^ Hepatotoxicity triggered by VEGF inhibitors is suspected to result from direct and/or indirect hepatotoxicity.^17^ Direct hepatotoxicity might arise from either the formation of a reactive metabolite or unintended multikinase inhibition, which could perturb various hepatocellular processes and lead to cell death.^18^ Indirect hepatotoxicity could ensue from the disruption of sinusoidal blood vessels' structure and function. A previous study reported hepatotoxicity secondary to the use of pazopanib in patients with renal cell carcinoma.^19^ A meta-analysis of genome-wide association studies suggested an increased risk of liver enzyme elevations in pazopanib-treated patients carrying the HLA-B*57:01 allele.^20^

In conclusion, we demonstrate a case with severe mixed hepatocellular and cholestatic DILI conforming to the Hy law, as evidenced by clinical improvement after axitinib discontinuation and exacerbated liver injury upon readministration. Physicians using axitinib should be aware of its potential for severe hepatotoxicity and remain cautious when using it combined with immunotherapy. Further studies are warranted to elucidate optimal management strategies and preventive measures.

## DISCLOSURES

Author contributions: YH Yeo wrote the manuscript. W. Ayoub, JD Yang, and A. Kuo revised the manuscript. HD Trivedi revised the manuscript and is the article guarantor.

Financial disclosure: None to report.

Informed consent was obtained for this case report.
